# Risk factors associated with the failure of secondary alveolar bone grafting with autologous iliac crest bone in patients with alveolar cleft defects: a systematic review

**DOI:** 10.3389/froh.2025.1640933

**Published:** 2025-11-11

**Authors:** Jiangyi Wu, Jun Zhuang, Yuan Ma, Lin Yin, Yongqian Wang

**Affiliations:** 1 Plastic Surgery Hospital, Chinese Academy of Medical Sciences and Peking Union Medical College, Beijing, China; 2 Maxillo-facial Surgery Center, Plastic Surgery Hospital, Chinese Academy of Medical Sciences and Peking Union Medical College, Beijing, China; 3 Center for Cleft Lip and Palate Treatment, Plastic Surgery Hospital, Chinese Academy of Medical Sciences and Peking Union Medical College, Beijing, China

**Keywords:** secondary alveolar bone grafting, alveolar cleft, failure, risk factors, bone grafting

## Abstract

**Purpose:**

This study aimed to perform a systematic review of the risk factors associated with secondary alveolar bone grafting (SABG) failure in patients with cleft alveolus.

**Methods:**

A comprehensive search was conducted across PubMed, Scopus, Embase, and Web of Science databases from their inception to 24 February 2025, to identify relevant studies. The search keywords included “alveolar cleft” combined with either “survival” or “failure.” Studies that investigated risk factors for the failure of SABG using autologous iliac crest bone were included in this review. Relevant data, including odds ratios, hazard ratios, or comparisons of variables between patients with and without SABG failure, were recorded and analyzed.

**Results:**

A total of nine studies, encompassing 1,855 grafts, were included. The most commonly used definition of SABG failure was Bergland grade 3 or 4. Reported failure rates varied significantly across studies, ranging from 1.0% to 45.1%. The primary risk factors for SABG failure included increased age at SABG (reported in four studies), poor oral hygiene (two studies), and the presence of an erupted lateral or canine tooth (three studies). Additionally, non-Caucasian ethnicity, international adoptee, large cleft size, a history of cleft lip/palate revision or oronasal fistula, nasoalveolar molding, and premaxillary osteotomy were also associated with a higher risk of SABG failure. No significant association was found between SABG failure and sex, alveolar cleft type (bilateral or unilateral), preoperative expansion, or preoperative orthodontics.

**Conclusions:**

The definition of SABG failure varied across studies, with Bergland grade 3 or 4 being the most commonly used criteria. The primary risk factors for SABG failure included increased age, poor oral hygiene, and the presence of an erupted lateral or canine tooth. Surgeons should be aware of these risk factors to optimize surgical strategies and guide patients effectively.

## Introduction

Alveolar clefts, frequently observed in individuals with cleft lip and palate, interrupt the continuity of the dental arch, adversely affecting both the functional integrity and aesthetic appearance of the oral cavity (1). The primary aims of treating alveolar clefts involve not only aesthetic improvement but also the restoration of functional integrity to the dental arch, facilitating the eruption of teeth and providing adequate support for the nasal base (2–4). Secondary alveolar bone grafting (SABG), utilizing autologous bone from the iliac crest, is widely recognized as the standard surgical intervention for repairing these defects, typically performed during the mixed dentition phase (5, 6). Despite progress in surgical techniques and biomaterials, the procedure continues to exhibit a variable failure rate, ranging from 5% to 68% (7), which underscores the need for a comprehensive understanding of the risk factors contributing to the failure of this procedure.

Numerous studies have identified risk factors associated with SABG failure, encompassing various aspects such as patient characteristics (7, 8) (e.g., age at the time of SABG), dental eruption status (9, 10) (e.g., presence of an erupted lateral incisor), alveolar cleft condition (8, 11) (e.g., cleft size), and history of prior interventions (9, 11) (e.g., preoperative orthodontic treatment). However, there is no consistent definition of SABG failure, and some studies have reported conflicting findings. Given the heterogeneity across studies and the limited clinical evidence, a systematic review is essential to comprehensively summarize the risk factors associated with SABG failure.

Accordingly, the primary aim of this study was to conduct a systematic review of the definition of SABG failure and identify its associated risk factors when using autologous iliac crest bone.

## Methods

This systematic review has been registered with the International Prospective Register of Systematic Reviews (PROSPERO, ID: CRD420250648819) and the results were reported according to the latest Preferred Reporting Items for Systematic Reviews and Meta-Analyses (PRISMA) guidelines (12).

### Search strategy and study selection

Studies were retrieved from PubMed, Scopus, Embase, and Web of Science from their inception to 24 February 2025, using the keywords “alveolar cleft” paired with either “survival” or “failure.” The detailed search strategies for each database are presented in Supplementary Table S1. The inclusion criteria were as follows: (1) studies investigating risk factors associated with the failure of SABG using autologous iliac crest bone; (2) a clear definition of SABG failure; and (3) published in English. The exclusion criteria included: (1) conference abstracts; (2) reviews and systematic reviews; (3) technical studies; and (4) animal or cadaveric studies. The study selection process was independently conducted by two reviewers (blinded for review). Any disagreements were resolved through discussion, with a third reviewer (blinded for review) making the final decision if consensus could not be reached. Titles and abstracts of all retrieved studies were carefully examined to exclude those clearly ineligible. The full texts of the remaining articles were then obtained and thoroughly screened to determine final eligibility.

### Quality assessment

The methodological quality of included studies was assessed according to the methodological index for non-randomized studies (MINORS) (13). Each study was scored across the 12 categories, with scores of 0 (not reported), 1 (reported but inadequate), or 2 (reported and adequate). The assessment was independently performed by two reviewers (blinded for review), and any discrepancies were resolved through discussion until a consensus was achieved.

### Data extraction

Basic information of studies, including the year of publication, study design, sample size, patient demographics, and mean follow-up period, was extracted. Additionally, key findings of an individual study on the risk factors for SABG failure were summarized. The potential risk factors investigated in individual studies and relevant data, such as odds ratios (ORs), hazard ratios, or comparisons of variables between patients with and without SABG failure, were collected. The results of the multivariate analysis were prioritized. The significant risk factors or the factors investigated by two or more studies were summarized.

### Statistical analysis

All statistical analyses were performed using R software for Mac (Version 4.3.2). Given the inconsistent reporting formats for the same variables and the limited number of available studies for a single variable, conducting a meta-analysis was challenging. Instead, the results were synthesized qualitatively, and the key findings were presented in a summary format. For variables with specific categories and their frequencies, univariate logistic regression was performed to obtain OR values and *P*-values.

## Results

**Figure 1:**
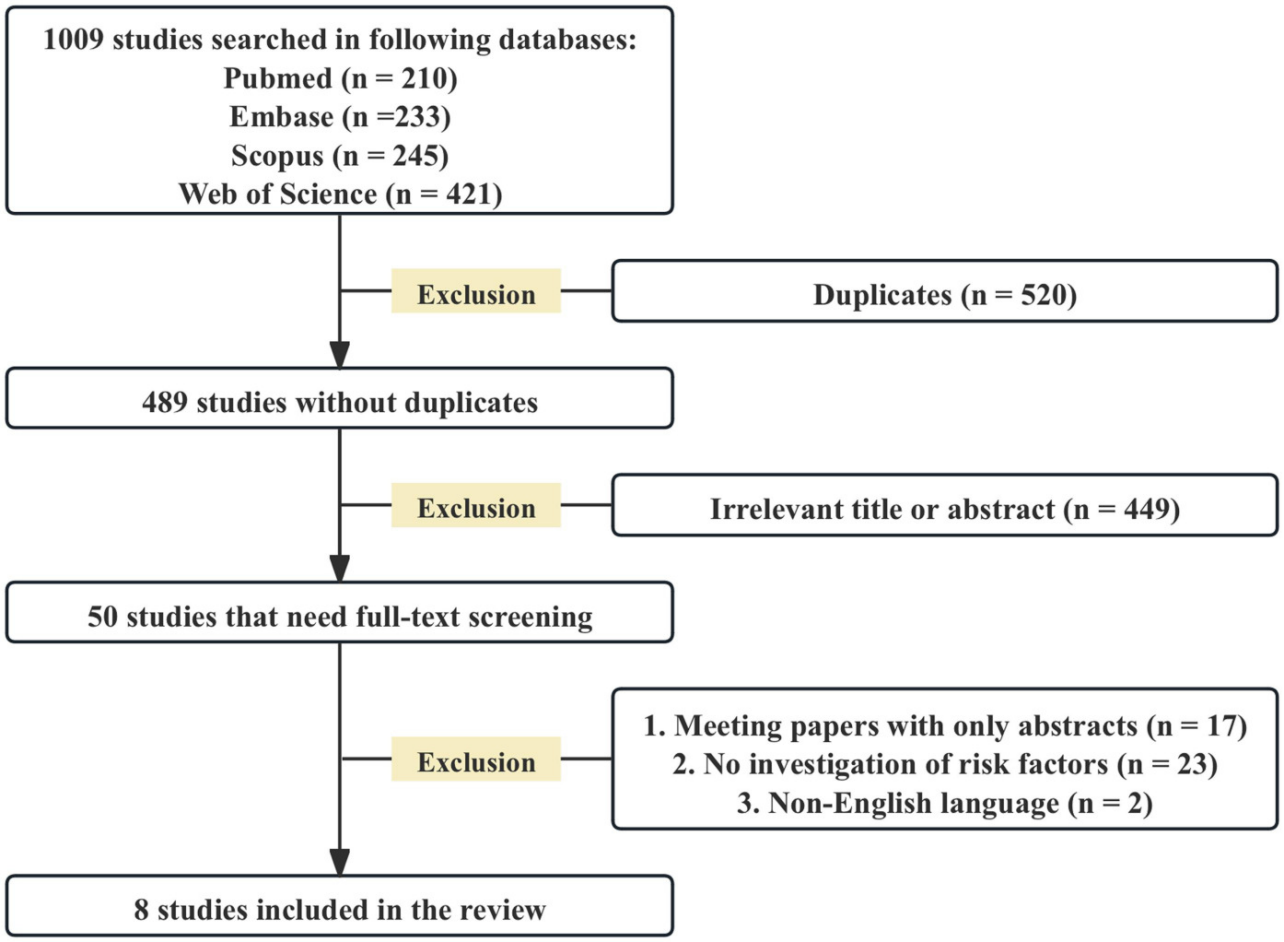
A PRISMA flowchart is employed to elaborate on the database searches, the quantity of abstracts screened, and the number of full - texts retrieved.

A total of 1,009 studies were retrieved from four databases. After removing 520 duplicates and excluding 449 studies based on title and abstract screening according to the inclusion and exclusion criteria, 50 studies remained. Then, a thorough full-text review was conducted. Ultimately, eight studies (7–11, 14–16) met the eligibility criteria and were included in the final review (Figure 1).

### Study characteristics

Table 1 present**s** the characteristics of the included studies, encompassing a total of 1,855 grafts with an average of approximately 204 patients per study (range: 49–900). The mean age at SABG varied from 8.1 to 12.4 years, with an overall average of 9.8 years across studies. The mean proportion of male patients was 58.0% (range: 53.0%–67.3%). All of the included studies utilized autologous iliac crest grafts. The follow-up period ranged from 3.0 months to 16.0 years.

**Table 1:** Study characteristics.

Study	Design	Sample size, *n*	Mean age at SABG, years	Male sex, %	Type of graft	Mean follow-up period
Williams (16)	Retrospective	157	10.3	54.9	Autologous iliac crest	NR
Toscano et al. (15)	Retrospective	49	10.3	67.3	Autologous iliac crest	1.9 years
Meyer et al. (9)	Retrospective	123	12.4	56.9	Autologous iliac crest	16.0 years
Lundberg et al. (10)	Retrospective	100	9.2	53.0	Autologous iliac crest	7.2 years
Kimia et al. (14)	Retrospective	195	8.1	56.8	Autologous iliac crest	3.6 years
Chalien et al. (8)	Retrospective	131	9.0	56.5	Autologous iliac crest	3.0 months
Smith et al. (11)	Retrospective	200	9.1	61.3	Autologous iliac crest	6.0 months
Padwa et al. (7)	Retrospective	900	9.9	59.3	Autologous iliac crest	8.8 months

NR, not reported; SABG, secondary alveolar bone grafting.

### Definition and prevalence of SABG failure

Bergland grade 3–4 was the most commonly used definition of failure [four studies (8–10, 16), ranging from 9.0% to 45.1%]. Additionally, failure was also defined by Bergland grade 2–4 or Witherow score < 1 (8.2%) (15), Bergland grade 4 (13.8%) (14), Kindelan grade 3–4 (1.0%) (11), and cone beam computed tomography (CBCT) observations of inadequate vertical bone height (≥50% root exposure), insufficient labiopalatal thickness (<75% root width), or poor nasal piriform symmetry (≥3 mm) (5.4%) (7) (Table 2).

**Table 2:** Comprehensive overview of results from individual studies.

Study	Definition of SABG failure	Incidence of SABG failure	Key findings on risk factors for SABG failure
Williams (16)	Bergland grade 3–4	45.1%	Non-Caucasian (OR = 5.42, *P* = 0.037)
			Per month increase in age (OR = 1.04, *P* = 0.007)
Toscano et al. (15)	Bergland grade 2–4 or Witherow score <1	8.2%	No significant risk factors found
Meyer et al. (9)	Bergland grade 3–4	17.9%	Increased age (comparing <9, 9–11, 11–14, and >14 years, *P* = 0.03)
			Distal tooth unerupted (OR = 3.47, *P* = 0.02)
Lundberg et al. (10)	Bergland grade 3–4	9.0%	Poor oral hygiene (OR = 11.39, *P* = 0.002)
Kimia et al. (14)	Bergland grade 4	13.8%	Age > 9 years (OR = 2.81, *P* = 0.048)
			International adoptee (OR = 4.26, *P* = 0.039)
			History of ONF (OR = 2.60, *P* = 0.015)
			History of NAM (OR = 0.20, *P* = 0.012)
			History of cleft lip/palate revision (OR = 5.64, *P* = 0.018)
Chalien et al. (8)	Bergland grade 3–4	17.5%	Age > 9 years (OR = 3.24, *P* = 0.029)
			Poor oral hygiene (OR = 4.22, *P* = 0.016)
			Eruption of Lateral or canine (OR = 5.50, *P* = 0.049)
Smith et al. (11)	Kindelan grade 3–4	1.0%	No significant risk factors found
Padwa et al. (7)	Inadequate vertical bone height (≥50% root exposure), or insufficient labiopalatal thickness (<75% root width), or poor nasal piriform symmetry (≥ 3 mm)	5.4%	Erupted canine (OR = 8.39, *P* < 0.001)
			Large bony cleft defect (OR = 3.64, *P* = 0.003)
			Premaxillary osteotomy (OR = 2.69, *P* = 0.038)
			History of failed bone graft (OR = 5.07, *P* < 0.001)

NAM, nasoalveolar molding; ONF, oronasal fistula; OR, odds ratio; SABG, secondary alveolar bone grafting.

### Quality assessment

All of the included studies were retrospective with a level of evidence of III. The mean MINORS score was 15.75 out of an ideal 24 (range: 15–18) (Table 3).

**Table 3:** Summary of quality assessment according to MINORS.

Study	Q1	Q2	Q3	Q4	Q5	Q6	Q7	Q8	Q9	Q10	Q11	Q12	Total, *n* (%)
Williams (16)	2	2	0	2	2	1	1	2	1	2	1	2	18 (75.0)
Toscano et al. (15)	2	2	0	2	2	2	0	0	0	2	1	2	15 (62.5)
Meyer et al. (9)	2	2	0	2	2	2	0	0	0	2	1	2	15 (62.5)
Lundberg et al. (10)	2	2	0	2	2	2	0	0	0	2	1	2	15 (62.5)
Kimia et al. (14)	2	2	0	2	1	2	1	0	0	2	1	2	15 (62.5)
Chalien et al. (8)	2	2	0	2	2	1	1	0	0	2	1	2	15 (62.5)
Smith et al. (11)	2	2	0	2	2	2	2	0	0	2	1	2	17 (70.8)
Padwa et al. (7)	2	2	0	2	2	2	1	0	0	2	1	2	16 (66.7)

Q1, a clearly stated aim; Q2, inclusion of consecutive patients; Q3, prospective collection of data; Q4, endpoints appropriate to the aim of the study; Q5, unbiased assessment of the study endpoint; Q6, follow-up period appropriate to the aim of the study; Q7, loss to follow-up <5%; Q8, prospective calculation of the study size; Q9, an adequate control group; Q10, contemporary groups; Q11, baseline equivalence of groups; Q12, adequate statistical analyses.

### Patient characteristics

*Age at SABG.* A total of four studies (8, 9, 14, 16) identified increased age at SABG as a risk factor for failure. Among them, two studies (8, 14) found that performing SABG after the age of 9 increases the risk of failure, with ORs of 2.81 and 3.24, respectively. The other two studies (9, 16) reported that increased age was significantly associated with SABG failure. The remaining four studies (7, 10, 11, 15) found no significant association between increased age and SABG failure (Table 4).

**Table 4:** Summary of patient characteristics associated with SABG failure.

Study	Age	Sex	Oral hygiene	Race
Williams, (16)	**Per month (OR** **=** **1.04, *P*** **=** **0.007)**	NR	NR	**Non-Caucasians (OR** **=** **5.42, *P*** **=** **0.037)**
Toscano et al. (15)	>10 years (*P* = 0.94)	Female (*P* = 0.08)	NR	NR
Meyer et al. (9)	**Increased age (*P*** **=** **0.03)**	Female (*P* = 0.25)	NR	NR
Lundberg et al. (10)	>110 months (OR = 0.28, *P* = 0.124)	Female (OR = 4.55, *P* = 0.071)	**Poor (OR** **=** **11.39, *P*** **=** **0.002)**	NR
Kimia et al. (14)	**>9 years (OR** **=** **2.81, *P*** **=** **0.048)**	Female (OR = 1.49, *P* = 0.281)	NR	**International adoptee (OR** **=** **4.26, *P*** **=** **0.039)**
Chalien et al. (8)	**>9 years (OR** **=** **3.24, *P*** **=** **0.029)**	NR	**Poor (OR** **=** **4.22, *P*** **=** **0.016)**	NR
Smith et al. (11)	≥11 years (*P* = 1.000)	NR	NR	NR
Padwa et al. (7)	≥12 years (*P* = 0.107)	Female (OR = 1.70, *P* = 0.072)	NR	NR

NR, not reported; OR, odds ratio; SABG, secondary alveolar bone grafting.

Bold values indicate statistically significant differences (*P* < 0.05).

*Sex.* Although females tended to exhibit a higher failure rate, sex showed no significant association with SABG failure across five studies (7, 9, 10, 14, 15), with ORs of 4.55, 1.49, and 1.70, respectively (Table 4).

*Oral hygiene.* Additionally, two studies (8, 10) found that poor oral hygiene was significantly associated with a higher failure rate of SABG (Table 4).

*Race.* Two studies reported that non-Caucasians (16) and international adoptees (14) significantly increased the risk of SABG failure (Table 4).

### Dental eruption status

A total of three studies (7–9) identified the dental eruption status as a risk factor for SABG failure. Among them, two studies (8, 9) reported that an erupted lateral or canine tooth at the time of SABG significantly increas**ed** the risk of failure, with ORs of 3.47 and 5.50, respectively. Another study (7) found that an erupted canine tooth (OR = 8.39) was significantly associated with SABG failure (Table 5).

**Table 5:** Summary of dental eruption status associated with SABG failure.

Study	Dental eruption status
Williams (16)	NR
Toscano et al. (15)	NR
Meyer et al. (9)	**Lateral or canine (OR** **=** **3.47, *P*** **=** **0.020)**
Lundberg et al. (10)	NR
Kimia et al. (14)	NR
Chalien et al. (8)	**Lateral or canine (OR** **=** **5.50, *P*** **=** **0.049)**
Smith et al. (11)	NR
Padwa et al. (7)	**Canine (OR** **=** **8.39, *P*** **<** **0.001)**

NR, not reported; OR, odds ratio; SABG, secondary alveolar bone grafting.

Bold values indicate statistically significant differences (*P* < 0.05).

### Condition of the alveolar cleft

*Type of alveolar cleft.* A total of seven studies investigated the association between the type of alveolar cleft and SABG failure. However, none of the individual studies found a significant association (Table 6).

**Table 6:** Summary of condition of the alveolar cleft associated with SABG failure.

Study	Type of alveolar cleft	Cleft size
Williams (16)	NR	NR
Toscano et al. (15)	NS (*P* = 0.84)	NS (*P* = 0.64)
Meyer et al.(9)	Bilateral (OR = 1.32, *P* = 0.552)	NR
Lundberg et al. (10)	Bilateral (OR = 0.54, *P* = 0.578)	NR
Kimia et al. (14)	Bilateral (OR = 1.94, *P* = 0.077)	NR
Chalien et al. (8)	Bilateral (OR = 1.89, *P* = 0.205)	NR
Smith et al. (11)	NS (*P* > 0.999)	NR
Padwa et al. (7)	Bilateral (OR = 0.80, *P* = 0.475)	**Large size (OR** **=** **3.64, *P*** **=** **0.003)**

NR, not reported; OR, odds ratio; SABG, secondary alveolar bone grafting; NS, not significant.

Bold values indicate statistically significant differences (*P* < 0.05).

*Cleft size.* Two studies explored the association between cleft size and SABG failure. One study identified a large cleft size (≥7.5 mm) as a significant risk factor, with an OR of 3.64. In contrast, the other study found no significant association between alveolar cleft severity and SABG failure (Table 6).

### History of prior intervention

*Preoperative expansion.* A total of three studies (7, 10, 14) investigated the association between a history of preoperative expansion and SABG failure. However, none of these studies found a significant association (Table 7).

**Table 7:** Summary of history of prior intervention associated with SABG failure.

Study	Preoperative expansion	Preoperative orthodontics	Other surgeries
Williams (16)	NR	NR	NR
Toscano et al. (15)	NR	NR	NR
Meyer et al. (9)	NR	Yes (OR = 4.25, *P* = 0.172)	NR
Lundberg et al. (10)	Yes (OR = 0.64, *P* = 0.524)	NR	NR
Kimia et al. (14)	Yes (OR = 0.98, *P* = 0.323)	NR	**Cleft lip/palate revision (OR** **=** **5.64, *P*** **=** **0.018)**
			**ONF (OR** **=** **2.60, *P*** **=** **0.015)**
			**NAM (OR** **=** **0.20, *P*** **=** **0.012)**
Chalien et al. (8)	NR	NR	NR
Smith et al. (11)	NR	Yes (OR = 1.46, *P* = 0.789)	NR
Padwa et al. (7)	Yes (OR = 1.29, *P* = 0.429)	Yes (OR = 1.22, *P* = 0.632)	**Failed bone graft (OR** **=** **5.07, *P*** **<** **0.001)**
			**Premaxillary osteotomy (OR** **=** **2.69, *P*** **=** **0.038)**

NAM, nasoalveolar molding; NR, not reported; ONF, oronasal fistula; OR, odds ratio; SABG, secondary alveolar bone grafting.

Bold values indicate statistically significant differences (*P* < 0.05).

*Preoperative orthodontics.* A total of three studies (7, 9, 11) investigated the association between a history of preoperative orthodontics and SABG failure. However, there were no significant associations found in these studies (Table 7).

*Other surgeries.* Two studies (7, 14) found that there was a significant association between cleft lip/palate revision (OR = 5.64) or failed bone graft (OR = 5.07) and SABG failure. Additionally, it was found that a history of oronasal fistula (ONF) (OR = 2.60) and premaxillary osteotomy (OR = 2.69) was associated with higher odds of SABG failure. In contrast, a history of nasoalveolar molding (NAM) was significantly associated with a lower risk of SABG failure (OR = 0.20) (Table 7).

## Discussion

The key finding of this systematic review was that the definition and prevalence of SABG failure varied across studies. The primary risk factors identified included increased age at SABG, poor oral hygiene, and the presence of an erupted lateral or canine tooth. Additionally, non-Caucasian ethnicity, international adoption, large cleft size, a history of cleft lip/palate revision or ONF, NAM, and premaxillary osteotomy were also found to be associated with a higher risk of SABG failure.

There was considerable variation in the definition of SABG failure across different studies. Currently, the primary criteria for evaluating postoperative outcomes of SABG were the Bergland classification (17), which was most widely accepted, long-established, and conveniently evaluated through assessing the graft height based on radiographic imaging. SABG failure was typically defined as Bergland grade 3 or 4, indicating that the grafted bone height is <75% of the normal alveolar height (18–20). And there were also other standards for assessing bone mass, such as the Enemark standard (21) and the Kindelan standard (22). Additionally, Padwa et al. (7) employed CBCT for a more comprehensive evaluation of the postoperative graft condition. These discrepancies in the definition may impact the overall understanding of SABG failure. Future studies are expected to adopt more widely accepted and standardized criteria (e.g., Bergland grade 3 or 4) for consistent evaluation.

Age at SABG was one of the most documented potential factors associated with the prognosis of SABG. All eight studies included in this review investigated the association between increased age and SABG failure. Grafting was ideally timed during the mixed dentition phase (typically 9–11 years old) (23). Consistent with this statement, two studies (8, 14) found that being over 9 years old significantly increases the risk of SABG failure. Additionally, two other studies (9, 16) also identified a significant association between increasing age as a continuous variable and SABG failure. The remaining studies did not find a statistically significant association between age and SABG failure, which may be due to the use of a higher threshold for age classification (10–12 years). The significant association between increased age and SABG failure is likely because bone healing capacity and regenerative potential diminish with age, and later grafts often coincide with more established scar tissue or less eruptive stimulation from adjacent teeth (24, 25). Notably, the interrelationships between age and dental eruption status, cleft severity, and prior surgical interventions also collectively contributed to the elevated failure risk in older patients. Future studies should account for similar interactive effects among these variables in their analyses. Furthermore, several studies (14, 16) included in this review identified a significant association between specific racial groups (non-Caucasians and international adoptees) and an increased risk of SABG failure. Given that the mechanisms underlying these associations remain poorly understood, further large-scale investigations are warranted to elucidate the potential biological and sociocultural factors contributing to this observed disparity.

Poor oral hygiene was also found to be associated with a higher risk for SABG failure. Inadequate plaque control and gingival health can lead to infection, flap dehiscence, or poor healing at the graft site, compromising the graft's integration (26). This finding underscored the importance of rigorous oral hygiene protocols before and after SABG (10). To minimize infection risk and enhance bone graft healing, patients should maintain optimal oral hygiene with the absence of active periodontal disease and significant plaque accumulation.

The presence of an erupted tooth was also a significant risk factor. Ideally, SABG was timed to occur before these teeth erupt, so that the graft can provide bony support for their eruption (6, 27). An erupted tooth in an ungrafted cleft was often associated with a persistent ONF and limited bone support, making subsequent grafting less likely to fully bridge the defect (28). As a result, in cases where patients present with an erupted canine, surgeons should counsel that the success rate of SABG may be significantly lower.

This is the first systematic review to summarize the risk factors for SABG failure. However, there was considerable heterogeneity among studies, including variations in the definition of SABG failure and the forms of different variables. As a result, only a qualitative systematic review was conducted, and a quantitative meta-analysis was not feasible. All of the studies included in this systematic review were retrospective in design, which inherently limits the strength of evidence for the reported outcomes. Additionally, this study included only factors reported in at least two articles and those that demonstrated statistically significant associations, making the results more concise and intuitive but potentially overlooking some information (like negative results) on account of the missing data. Controlling for confounding factors also posed a significant challenge, as only a subset of studies performed multivariate analysis. To minimize bias as much as possible, we selectively incorporated results from multivariable analyses.

## Conclusion

The definition of SABG failure varies across studies, with Bergland grade 3 or 4 being the most commonly used criterion. The key risk factors for SABG failure include increased age, poor oral hygiene, and the presence of an erupted lateral incisor or canine. Given the inherent limitations of including only retrospective studies in this systematic review, future high-level evidence studies remain warranted.

## Data Availability

The original contributions presented in the study are included in the article/Supplementary Material; further inquiries can be directed to the corresponding author.
