# Supplementary material for: Risk factors associated with the failure of secondary alveolar bone grafting with autologous iliac crest bone in patients with alveolar cleft defects: a systematic review
Source: Front Oral Health. 2025 Nov 11;6:1640933. doi: 10.3389/froh.2025.1640933 (PMC12644006; doi:10.3389/froh.2025.1640933)
Supplement: Supplementary file 1 [file Table1.docx]

**Table S1 Summary of Search Strategy**

| Database | Search Strategy |
| --- | --- |
| Pubmed | (alveolar cleft) AND (survival OR failure) |
| Scopus | TITLE-ABS-KEY ( ( alveolar AND cleft ) AND ( survival OR failure ) ) |
| Embase | alveolar AND cleft AND ('survival'/exp OR survival OR 'failure'/exp OR failure) |
| Web of Science | ( alveolar AND cleft ) AND ( survival OR failure ) (Topic) |
